# Smart Sensing of Pavement Temperature Based on Low-Cost Sensors and V2I Communications

**DOI:** 10.3390/s18072092

**Published:** 2018-06-29

**Authors:** Jorge Godoy, Rodolfo Haber, Juan Jesús Muñoz, Fernando Matía, Álvaro García

**Affiliations:** 1 Centre for Automation and Robotics (UPM-CSIC), Universidad Politécnica de Madrid, Calle José Gutiérrez Abascal, 2, 28006 Madrid, Spain; fernando.matia@upm.es; 2 Centre for Automation and Robotics (UPM-CSIC), Consejo Superior de Investigaciones Científicas, Ctra. de Campo Real, km 0.200, Arganda del Rey, 28500 Madrid, Spain; rodolfo.haber@csic.es (R.H.); alvaro.garcia.r@car.upm-csic.es (Á.G.); 3 GEOCISA Geotecnia y Cimientos S.A, Los Llanos de Jérez, 10-12, 28820 Coslada, Madrid, Spain; jmunozn@geocisa.com

**Keywords:** pavement monitoring, vehicle-to-infrastructure, wireless sensor network, modeling, multilayer perceptron

## Abstract

Nowadays, the preservation, maintenance, rehabilitation, and improvement of road networks are key issues. Pavement condition is highly affected by environmental factors such as temperature and humidity, hence the importance of building databases enriched with real-time information from monitoring systems that enable the analysis and modeling of the road properties. Information and communication technologies, and specifically wireless sensor networks and computational intelligence methods, are enabling the design of new monitoring systems. The main goal of this work is the design of a pavement monitoring system for measuring temperature at internal layers. The proposed solution is based on low-cost and robust temperature sensors, vehicle-to-infrastructure communications, allowing one to transmit information directly from probes to a moving auscultation vehicle, and a neural network-based model for prediction pavement temperature. User requirements drive probes’ design to a modular device, with easy installation, low cost, and reduced energy consumption. Results of the test and validation experiments show both the benefits and viability of the proposed system, which reflect in an accuracy improvement and reduction in routine test duration. Finally, data collected over a year is applied to assess the performance of BELLS3 models and the suggested neural network for predicting pavement temperature. The dynamic behavior of the predicted temperature and the mean absolute error of the neural network-based model are better than the BELL3 model, demonstrating the suitability of the proposed pavement monitoring system.

## 1. Introduction

Design and quality of road networks have been proven to be critical factors in the socio-economic development of any region [1,2,3]. Building a durable road requires pavement layers to be selected for the expected traffic and weather conditions, hence the interest in collecting critical data about pavement performance at in-service roads, allowing researchers to learn about how different materials respond to different traffic conditions and when and why failures occur [4]. Moreover, routine testing protocols are key for monitoring the state of internal pavement layers and planning maintenance services at the right moment, leading to more cost-effective roads [5,6,7]. The progress on sensoring systems and on information and communication technologies has favored the development of new methods to carry out pavement inspection.

In this sense, visual inspection and pavement auscultation is conducted by road administrators periodically. Although several parameters reflect pavement’s structural condition, surface deflection is the most common measurement. This term comes from latin word “deflecto”, which means “to bend”, referring to the recovered elastic vertical strain that is produced on pavement due to a load [8]. Depending on the type of pavement, two devices for nondestructive tests are mainly used: a falling weight deflectometer (FWD) and a curviameter [9,10,11]. The first one uses a dynamic load for delivering a transient impulse or impact to the pavement surface and measures the resultant response. On the other hand, a curviameter uses a vehicle with a high load on the rear axle and a series of sensors to measure the temporary deformation of the road surface when the rear axle is passing over.

As is mentioned in [8], environmental conditions such as humidity and temperature of the pavement influence deflection measurement, so normally it is necessary to correct or standardize the results. In this sense, pavement surface temperature is usually measured by infrared thermometers along the deflection test. However, the surface temperature is also affected by other conditions such as shadows, wind, and cloudiness, among others. For this reason, some protocols require measuring temperature at different depths, making the process more complex. In addition, for more detailed tests, temperature gradient of a pavement section is required.

Indeed, among the most useful arsenal of technologies, wireless sensor networks (WSNs) and computational intelligence methods have demonstrated their ability to monitor complex large scale systems, such as road networks [12,13]. Nowadays, WSNs are a breakthrough technology in areas such as home automation, automotive, agriculture, industry, and environmental. These networks consist of nodes based on small devices with sensing capabilities that collect and transmit information to a central device wirelessly. Most common applications are designed to sense environmental conditions such as temperature, sound, pollution, and so on [14,15]; however, its versatility allows for the implementation of this technology in a wide range of applications, such as traffic monitoring and vehicle-to-infrastructure communication (V2I) [16,17]. Artificial neural networks (ANNs), specifically the Multilayer Perceptron (MLP), the pioneering and most applied topology world-wide among ANNs, have received special attention.

The main contribution of this paper is the design and implementation of a pavement monitoring system based on low-cost and robust temperature sensors, WSNs, and an MLP for predicting pavement temperature. The proposed solution implements a communication scheme inherited from WSNs, where the nodes are temperature probes embedded on pavement. Probes were designed to reduce the duration of routine tests and improve measurement accuracy. In addition, by continuously monitoring the road, a more complete characterization of the pavement deflection can be provided. The remainder of the article is organized as follows: Section 2 presents an introduction to the modeling techniques applied for pavement monitoring. Section 3 provides an overview of the proposed monitoring system based on WSNs. The validation of the proposed system is presented in Section 4. Section 5 presents the results for temperature prediction on pavement. Finally, Section 6 draws some concluding remarks and future works.

## 2. Modelling Pavement Temperature

Since 1987, several highway agencies and research institutions have been collecting and evaluating data about the performance of different pavement sections across the United States and Canada, under the framework of the Long-Term Pavement Performance (LTPP) program. Initially, the LTPP program was launched as part of the Strategic Highway Research Program, a five-year applied study focused on the analysis of why some pavements perform better than others. However, since 1992, the LTPP program has continued operating under the Federal Highway Administration with an estimated $2.5 billion in savings for highways [4].

Within the LTPP, the Seasonal Monitoring Program (SMP) was designed to study the effect that seasonal variations have on pavement performance. The goal was to establish a relationship between pavement performance response measures such as deflection and profile, and temperature and season, as appropriate. From the data collected under the SMP framework, two models for temperature prediction at internal layers were presented by [18] BELLS2 and BELLS3.

**Figure 1:**
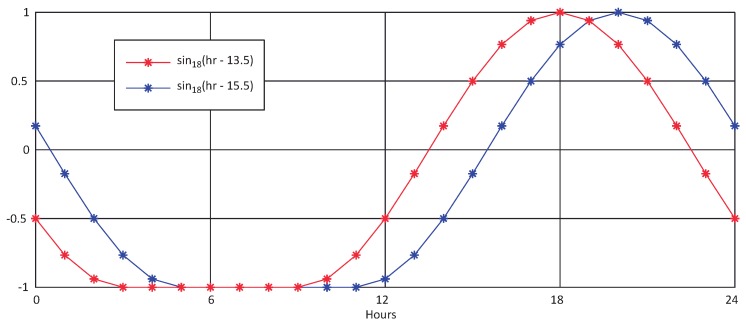
$sin_{18}$ functions along a day.

These models are evolved from the original BELLS model [19], relating the internal pavement temperature at a given depth with the average air temperature on the previous day and the measured surface temperature. The main difference between both models is the testing protocols to be applied with each one, the LTPP protocol or the routine FWD protocol. Details on each model can be found in [18]. Both models share the same equation, with different coefficients:

(1) $$T_{d}=A_{1}+A_{2}T_{S}+\left(\log_{10}\left(d\right)+A_{3}\right)\left(A_{4}T_{S}+A_{5}T_{A}+A_{6}\sin_{18}\left(h-15.5\right)\right)+A_{7}T_{S}\sin_{18}\left(h-13.5\right)$$

where *d* is the depth at which material temperature is to be predicted in mm, $T_{S}$ is the temperature on the surface in °C, $T_{A}$ is the average air temperature on the previous day in °C, *h* is the time of the day in a 24-h clock system, and $\sin_{18}$ is a sine function on an 18-h clock system, with 2π radians equal to an 18-h cycle. Coefficients for each model are shown in Table 1. Figure 1 plots both $sin_{18}$ functions.

### Multilayer Perceptron

The MLP is one of the most applied topologies of ANNs for modeling and pattern recognition [20,21,22]. An MLP can have one or more hidden layers [23]. Some works already reported in the literature have demonstrated that a single hidden layer is enough for representing complex processes [24]. For the sake of simplicity, considering only one hidden layer with a hyperbolic tangent activation function *H*, and a linear activation function, *L*, at the output, can be represented by

(2) $${\hat{y}}_{i}\left(w,W\right)=L_{i}\left[\sum_{j=1}^{Q}W_{ij}H_{j}\left(\sum_{k=1}^{M}w_{jk}u_{k}+w_{j0}\right)+W_{i0}\right]$$

where *Q* is the number of output neurons, *M* is the number of neurons in the hidden layer, *u* represents the inputs, and $\hat{y}$ is the output of the network. The weights are specified by the matrices w (input-to-hidden layer weights) and W (hidden-to-output layer weights).

Although a single hidden layer might be enough for some applications, it requires the input to be continuous and representable within a compact space. Thus, in order to capture high-order statistics, a greater number of hidden layers are needed. In this work, we propose a two-hidden-layer network for the estimation of internal pavement temperature. Two layers add levels of abstraction that cannot be as simply contained within a single layer of the same number of parameters. In a few words, two layers mean more non-linearities applied to the data.

## 3. System Overview

**Figure 2:**
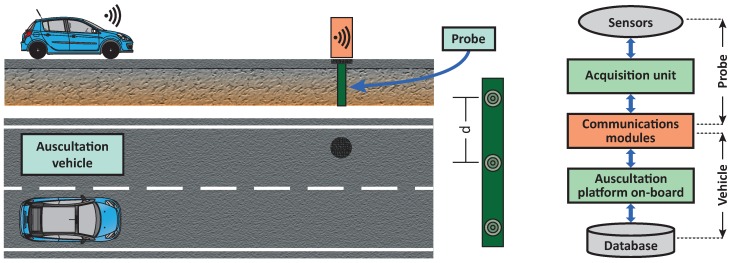
Conceptual design of the proposed monitoring system.

The design of the monitoring system responds to a main requirement: to reduce the duration of a routine test while improving the accuracy of temperature measurements in the pavement’s internal layers. Bearing this in mind, a system based on embedded probes and wireless communication is proposed (see Figure 2). Probes are designed to be installed by road administrators on key locations, measuring pavement temperature at several depths continuously. On the other hand, a platform installed on the auscultation vehicle collects the information from nearby probes on demand using V2I communication. Depending on the testing protocol, probes transmit either the most recent measurement or a set of recorded data.

In relation to V2I communication, a scheme based on WSN technology (802.15.4) is proposed. In contrast to systems based on 802.11p, which are expensive (thousands of dollars), commercial WSN solutions are widely applied nowadays and range from a few to hundreds of dollars, which influences the final cost of the monitoring system. Moreover, the authors’ previous works with both technologies demonstrated that WSN communications is more suitable for this application [17,25]. For example, thanks to the low power consumption of WSN nodes, probes would have a low maintenance cycle, since they can last more time powered by batteries and solar panels (>5 years).

Today’s standards for V2I communications do not consider pavement information within their payload [26]. Thus, probes’ data is transmitted using messages customized for each operation mode. On the following subsections, the details about the probes and the auscultation platform are presented.

### 3.1. Probes

**Figure 3:**
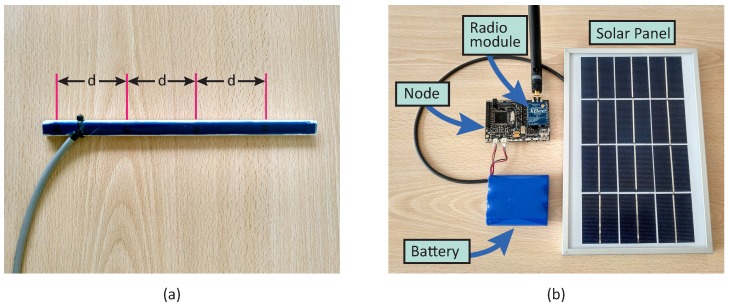
Probes’ hardware: (**a**) DS18B20 installed on frame; (**b**) WSN-node and power supply.

Figure 3 shows the probes that are composed of two main components: temperature sensors and a WSN node. The node collects data from sensors periodically, remaining in a stand-by state until a transmission order is received. In this sense, two operation modes are proposed: (i) instant measurement and (ii) data collection. For the first mode, the probe would await the auscultation order to measure and transmit pavement temperature. On the second mode, measurements would be stored on an internal database and then transmitted to the vehicle when demanded. Depending on the amount of data transmitted, the operation mode would condition the speed of the auscultation vehicle.

In order to validate the viability of the proposed system, a set of prototype probes were developed and installed in the facilities of the Centre for Automation and Robotics in Madrid, Spain. Each prototype was build using a Libelium Waspmote v1.1., with a XBee-Pro 802.15.4 EU communication module. This platform was selected from the previous experience on the development of WSN-based solution for traffic environments [17]. Temperature is measured using DS18B20 digital sensors, widely used in agricultural applications for soil monitoring. These sensors have an input range from −55 to 125 °C, with 0.5 °C accuracy and a programmable resolution from 9 to 12 bits (0.5 to 0.0625 °C).

Sensors are placed over a frame 5 cm apart from each other, measuring temperature from the surface to a 15 cm depth. This simplifies the installation or replacement of the sensors on pavement. Moreover, the frame is covered by a special resin that guarantees the contact with the pavement and protects the sensors. Finally, probes are powered by a Li-ion battery (6600 mAh, 3.7 V) and a small solar panel (234×160×17 mm, 7 V, 500 mA).

Sensors communicates over a 1-Wire bus, requiring only one data line for communication with the microcontroller on the node. Thus, the length of the cable required on the installation is minimized. A unique 64-bit serial code identifies each sensor on the bus. Depending on sensor resolution, the acquisition time ranges from 93.75 to 750 ms for 9 and 12 bits, respectively. Therefore, reading four sensors sequentially traduces in a total time from 375 ms to 3 s. Therefore, the maximum speed of the auscultation vehicle depends on probe configuration since the greater the resolution, the longer the vehicle must be near the probe. On the other hand, external interferences on the road could produce communication gaps, requiring the vehicle to circulate at slower speed.

**Figure 4:**
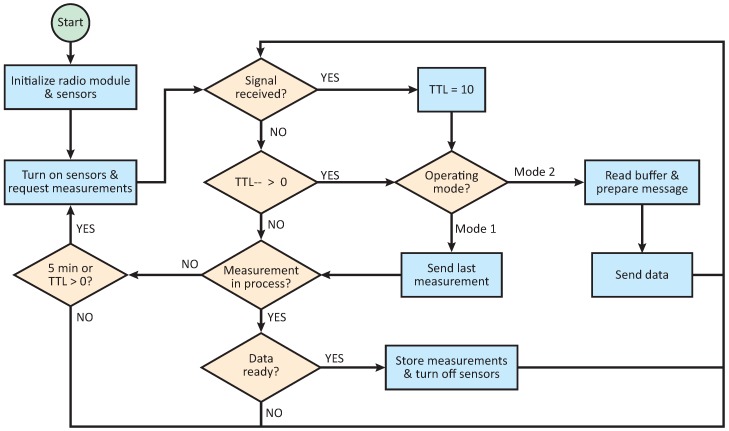
Probes operating scheme.

The operating scheme of the probes is presented on Figure 4. As is shown in the diagram, pavement temperature is captured every 5 min, the sampling time appropriate for the rate of change of the temperature (slow dynamic). All measurements are stored in an internal database that works as a circular buffer, replacing the oldest data. The size of the buffer depends on maximum device memory and probe configuration. When the auscultation signal is received, the operating mode is identified and the transmission started. If the instant measurement is requested, the probe first sends the last measurement stored on the database and then proceeds to read the sensors, transmitting the updated information as soon as it is available. Thus, in the worst case scenario, when the communication window is smaller than the sensors’ acquisition time, a 5-min age measurement is received by the vehicle. In order to ensure the quality of the data transmission, the probes are configured to transmit each message up to ten times at a 2 Hz rate or while the auscultation signal is received.

In case the auscultation system requests the entire database, the probe sends the data continuously, starting from the newest values. Data collection modes were not designed for moving vehicles, so in this mode the vehicle must remain inside the communication area until transmission is finished so that data loss does not occur.

Finally, it is important to remark that, once an order is received, the WSN communication is configured to answer only to the auscultation platform, meaning no data would be sent to any other device. The main rationale is to avoid data sniffing from possible external entities.

### 3.2. Auscultation Platform

From a communication view point, probes remain in a stand-by state until a signal is received. Being so, the vehicle must act as a moving beacon, activating the probes as they enter in a communication range. While it is true, a location system could be used for detecting the best location to send the signal, this would require a GNSS equipment and a digital map with the probe’s position, increasing the final cost and complexity of the monitoring system. Moreover, unpredictable factors such as traffic, obstacles, and meteorological conditions could reduce the communication range, so a location-based system would not guarantee that the signal is received by the probe.

**Figure 5:**
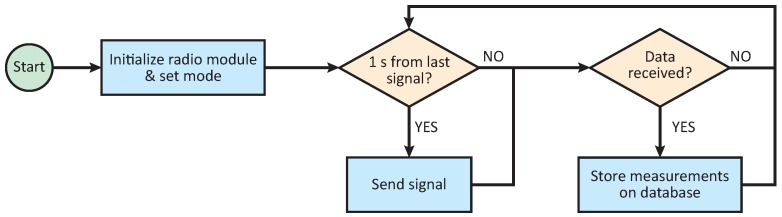
Operating Scheme for the Auscultation Platform.

Bearing this in mind, the operating mode of the auscultation platform is presented in Figure 5. It can be inferred from this figure that the platform sends promiscuous requests periodically until a probe is detected. Once the first data package is received, the platform switches to record mode, awaiting a user-configurable time before restarting the beacon transmission. Note that, for instant measurements, there is no need for the vehicle to stop. However, for collecting the entire database, the vehicle must remain within the communication range of the probe until all data is received.

With the aim of validating the proposed auscultation platform, a prototype was developed and embedded in a vehicle. The platform is based on a WSN Gateway also provided by Libelium. This device enables any computer to communicate with the WSN thought a USB interface. From the computer side, the data is sent and received transparently, without packaging or unpackaging into a WSN message. Auscultation software was developed in C++ on an Ubuntu OS. However, the simplicity of the design makes it possible to adapt the software to any other language or platform.

## 4. System Validation

Once the prototypes of the probes and the auscultation platform were developed, a set of tests were carried out to demonstrate the viability of the monitoring system and the proposed operating modes. Experiments for each operating mode are presented in the following subsections.

### 4.1. Mode 1: Instant Measurement Validation

**Figure 6:**
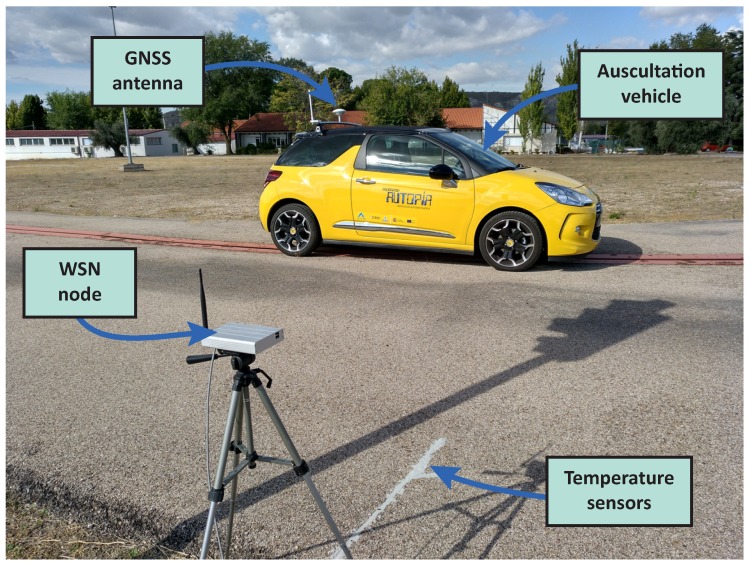
Auscultation vehicle and probe prototype on a test road.

In order to evaluate the performance of the instant measurement mode, a probe was installed on the test track of the Centre for Automation and Robotics and a prototype vehicle equipped with the auscultation platform. Figure 6 shows how an RTK-GNSS receiver is also installed on the vehicle. This device provides vehicle location at a 20 Hz rate and a 5 cm accuracy using corrections from a reference station within CAR’s facilities. The vehicle location was used for determining the maximum range of the system.

Results of performance tests carried out with free line-of-sight shown modules are able to communicate at distances up to 330 m without a loss of messages. At larger distances, some data packages are not delivered and the performance of the system is compromised. On the other hand, for the worst case scenario (line-of-sight with obstacles), a communications range up to 90 m without loss of packages was found. Taking into account the normal speed for an auscultation vehicle is around 5 m/s ( 20 km/h), in the worst case, the monitoring system has up to 18 s to establish communication. This time window is enough for transmission of the instant reading from the probe, considering a message is transmitted within hundreds of milliseconds [17].

### 4.2. Mode 2: Data Collection

In order to analyze the performance on continuous data collection, two probe prototypes were permanently installed on two different pavements of the test track and temperature measured over a year (August 2016–August 2017). A stationary auscultation platform was installed inside the control booth of the track, emulating an auscultation vehicle in a static position. The probes’ data was collected once a day along with meteorological information from a nearby station (air temperature, rain, wind, etc.). All registered measurements were combined into a database for further analysis of temperature diffusivity on pavement (see Section 5).

**Figure 7:**
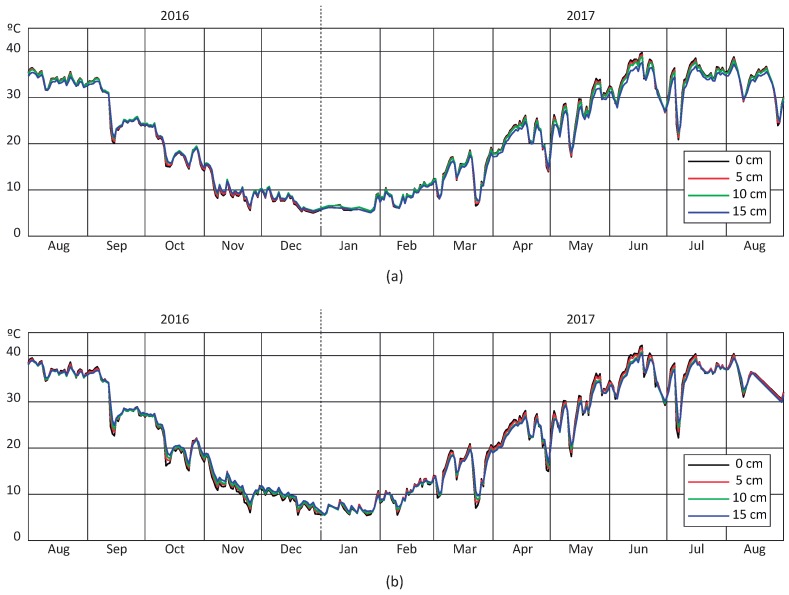
Evolution of daily average temperature along the year: (**a**) Probe A (**b**) Probe B.

Figure 7 depicts the behavior of daily average temperature for each probe over a year. It is clearly illustrated that the average temperature evolves as expected for each sensor, reaching the minimum and maximum values around January and June, respectively. Moreover, despite being only 2 m apart from each other, a slight difference can be appreciated among temperature evolution in each probe. This is explained by the different pavement materials.

**Figure 8:**
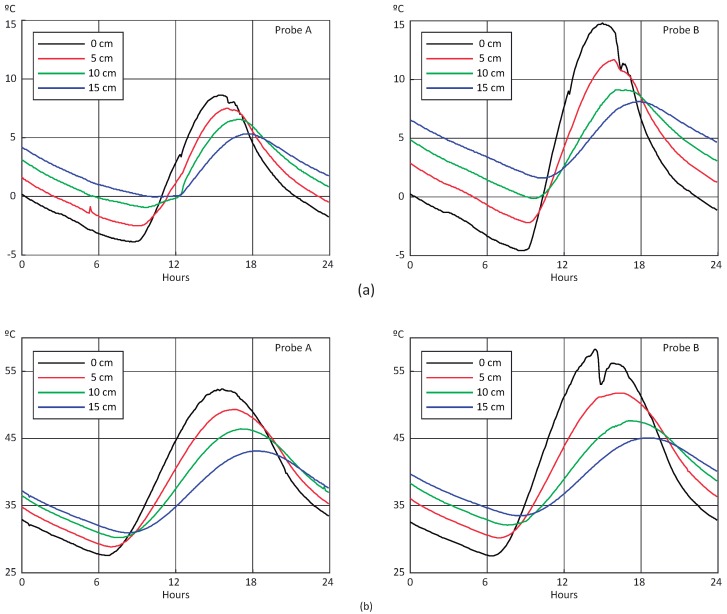
Evolution of temperature measurements per sensor over a day: (**a**) winter, 18 January 2017; (**b**) summer, 16 July 2017.

A day-view for each probe is shown in Figure 8. Top and bottom graphs depict temperature fluctuation over one winter day and one summer day, respectively. The largest difference appears at the surface level for both scenarios, with Probe A presenting a narrow range. The peaks observed in the Probe B graphs around 15–16 h correspond to the shadow of a traffic light. Note that, as the depth increases, the amplitude of temperature fluctuation decreases and the maximum and minimum values phase out. This is due to the thermal inertia of the pavement.

## 5. Pavement Modeling

After having validated the probe functionality for the two operating modes, data collected for one year with pavement and meteorological measurements were used for two additional tasks: (i) verification of the BELLS3 model and (ii) the design and training of the MLP. Details of each tasks are described in the next subsections.

### 5.1. BELLS Model

BELLS models are one of the most used techniques applied for estimation of pavement temperature at internal layers. Utilization of the appropriate model enables one to calculate the temperature within an asphalt pavement at each location using a temperature sensor mounted on the auscultation vehicle. Surface measurements can be obtained quickly, eliminating the need of drilling holes on the pavement, which is time-consuming and may alter the measurements due to the heat of drilling [18].

From the data collected by the probes installed at CAR’s facilities and the nearby meteorological station, a verification of the BELLS3 model was carried out. This model was adjusted for routine tests and allows one to estimate temperature at depth *d* from temperature at surface ($T_{S}$), time of day (*h*), and average air temperature of the previous day ($T_{A}$) (see Section 2).

**Figure 9:**
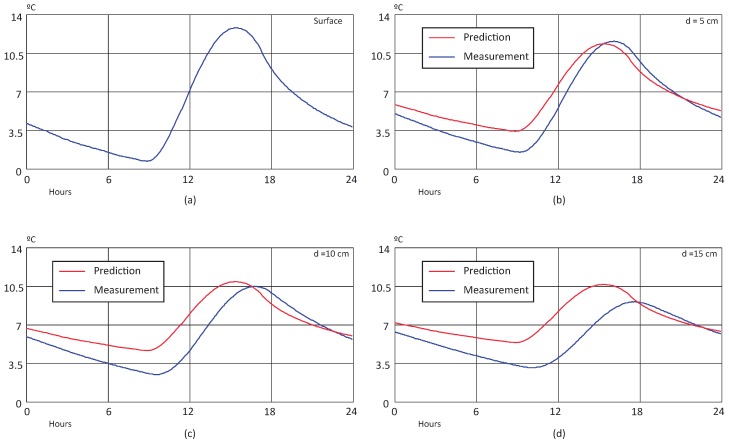
BELLS3 prediction over a winter day: (**a**) surface temperature, (**b**) temperature at 5 cm, (**c**) temperature at 10 cm, and (**d**) temperature at 15 cm.

**Figure 10:**
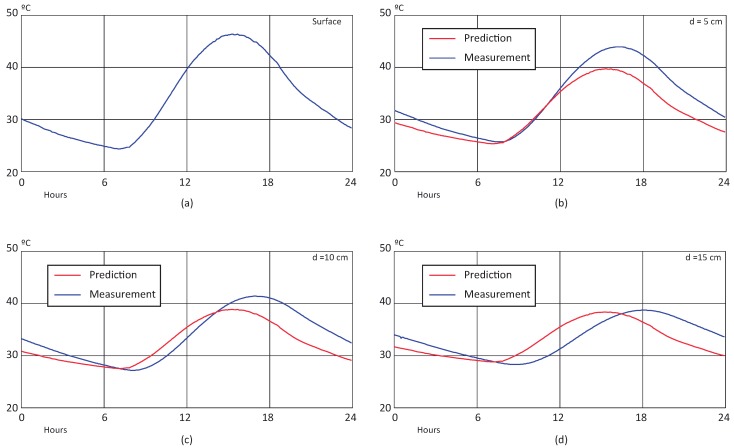
BELLS3 prediction over a summer day: (**a**) surface temperature, (**b**) temperature at 5 cm, (**c**) temperature at 10 cm, and (**d**) temperature at 15 cm.

Figure 9 and Figure 10 shows the results of the BELLS3 prediction for winter and summer scenarios. The input surface temperature over a day is shown in (a), while (b–d) illustrate the behavior of predicted (estimated by the model) and real (measured) temperatures at 5, 10, and 15 cm depths, respectively. The BELLS3 model’s estimation approximates the real value with large error for all cases. Moreover, it is unable to reflect the phase-out caused by the thermal diffusivity of the material at deeper levels.

In order to perform a comparison in both scenarios, the Mean Average Percentage Error (MAPE) was calculated for each case, and the results are summarized in Table 2. It is shown how, in the winter scenario, the BELLS3 model has a poor performance due to the low temperature of the pavement and the large error on the estimation. On the other hand, for the sample corresponding to a summer day, the model has a better performance. This behavior can be explained by the offset appreciated between the prediction and measurement in the winter graphs (see Figure 9).

### 5.2. MLP Training and Validation

Certainly, the BELLS3 model’s verification yielded a poor performance for temperature prediction at internal layers. This behavior is due to the non-linearities of the pavement temperature. From a simple system view point, road pavement, as any other soil, acts as a thermal accumulator, storing thermal energy received from the sun as radiation [27]. Nevertheless, meteorological conditions such as clouds, wind, and rain induce perturbations on the system that are hard to estimate or model. Furthermore, sun radiation depends on the geographical location and the time of the year, which are not considered as input on the original model. Therefore, to overcome the limitations of BELL2 and BELL3 models, a model for estimating temperature based on an ANN is proposed in this work.

In order to keep the system as simple as possible, the same inputs as for BELLS models are considered for the MLP model, i.e., the hour of the day, the average air temperature on the previous day, and the temperature measurement at the surface and depth at which the temperature is needed. In addition, the day of the year is also included as a fifth input in a 366-day format. By this way, this fifth variable reflects changes in the amplitude of sun radiation.

**Figure 11:**
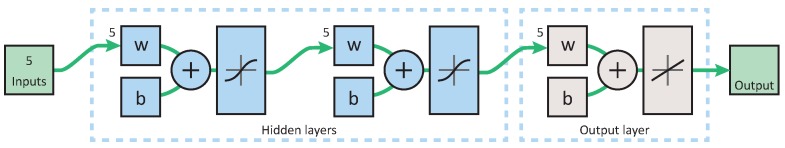
Diagram of the multilayer perceptron for temperature prediction.

Taking into account the non-linearities and uncertainty, a multilayer perceptron with two hidden layers is proposed. As is shown in the network diagram (Figure 11), the number of neurons for each hidden layer is the same as the number of inputs of the system. This choice serves to guarantee enough degrees of freedom for representing relationships among all the input variables. The Levenberg–Marquardt technique was selected as a training method because this method has demonstrated to be more powerful than the conventional gradient descent techniques [28].

Firstly, the collected data were filtered in order to remove days with abnormal behavior. Data flagged as abnormal correspond to those days where the sun radiation is especially low due to heavy clouds and/or rains. Once filtered, only 60% of the data was used for network training, while the remaining data were equally divided on test and validation datasets. All datasets were taken from real data sampling randomly within 4 seasonal clusters, guaranteeing that each one counted with equally distributed data over the year. Despite appearing to be a short overall measurement time for reflecting pavement behavior before all possible situations, the 1-year dataset includes enough information for assessing the viability of the proposed approach.

**Figure 12:**
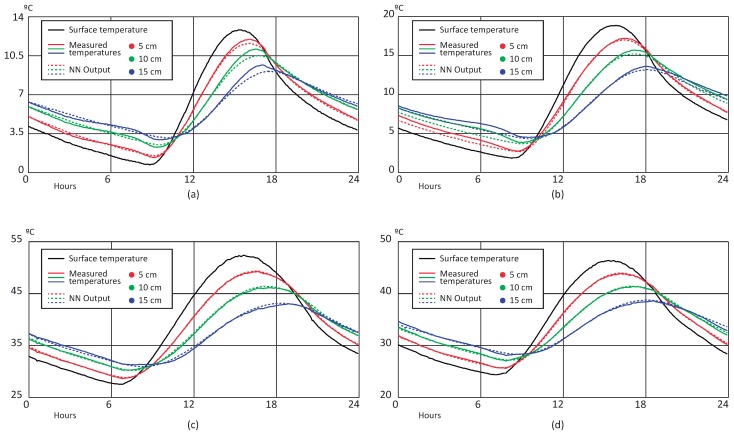
Measured temperature vs. neural network (NN) prediction: (**a**) winter scenario—test data; (**b**) winter scenario—validation data; (**c**) summer scenario—test data; (**d**) summer scenario—validation data.

Figure 12 shows a sample of the estimation results obtained after training the MLP. Images (a) and (b) correspond to winter days from test and validation datasets, respectively. Likewise, images (c) and (d) plot the temperature estimation for summer days from test and validation datasets. In all figures, dotted lines represent the estimated temperature (i.e., MLP output) and the continuous lines represent real measured temperature. Moreover, each depth is plotted with different colors.

**Figure 13:**
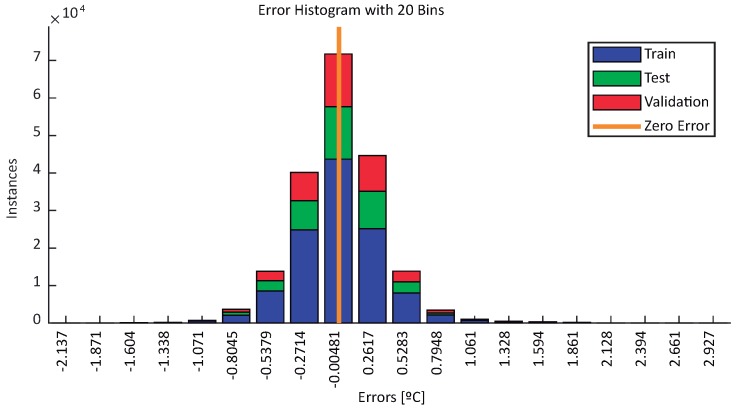
Error distribution for training, test, and validation datasets.

Comparing these results with the ones obtained for the BELLS3 estimation by visual inspection, it is clearly demonstrated that the MLP has a better response. For all cases, the estimation approximates the real measured temperature with less error than BELLS3, especially on the sunlight hours (between 10 and 19 h on winter days and 8 and 21 h on summer days). Overall, temperature estimation based on the BELLS3 approach showed a worse behavior. Although the estimation was close to the measurement, the observed error was higher than 5% on summer days and 35% on winter days. Table 3 summarizes the MAPE obtained for all scenarios. As for the BELLS model, the larger errors are obtained in the winter scenarios. This behavior, as well as the estimated errors depicted in pictures before sunrise time, might be due to a higher influence of disturbances and external variables not considered as model inputs, such as current air temperature, wind speed, and soil moisture. Nevertheless, according to Figure 13, the estimation error of the MLP is less than 1 °C for almost all samples included in the three datasets.

## 6. Conclusions and Future Works

In this work, a monitoring system of road pavements based on low-cost and robust temperature sensors was designed and implemented. The proposed solution is based on temperature probes with V2I communications capabilities, designed to be embedded on key points of the pavement by road administrators. Thus, auscultation vehicles are able to measure temperature at several depths without the need of stopping for drilling and measuring directly on the pavement. Besides improving the accuracy of the measurements, the system also contributes to reducing the duration of routine tests.

The performance of the proposed system was evaluated by the permanent installation of two prototypes in the test track of the Centre for Automation and Robotics in Madrid, Spain. Likewise, a prototype of auscultation was developed for testing the V2I communication capabilities. Results obtained during 1-year tests demonstrate the viability of the proposed solution for road monitoring applications. Moreover, data collected during the test were used for verification of the BELLS3 model and the development of a neural network-based model for predicting temperature pavement. The study, supported by a year’s worth of data, demonstrates that the MLP outperforms the traditional model for estimating pavement temperature, showing a better performance than the BELLS model. Moreover, the study also corroborated that the deeper the layer, the worse the performance of the model since it is unable to reflect the phase-out of the signal caused by the thermal diffusivity of the road, which depends on pavement materials.

Future research will be focused on extending these results by including material parameters as input to the model. Thus, the model can be extended to other pavement materials. Moreover, the study of the influence of input variables on thermal response will be conducted for analyzing the need of new inputs inclusion or removal. Other computational intelligence techniques will be explored to improve the model accuracy. Finally, the installation of probes on roads open to traffic is under negotiation with road administrators.

## Figures and Tables

**Table 1:** Coefficients for BELLS2 and BELLS3 models.

	$A_{1}$	$A_{2}$	$A_{3}$	$A_{4}$	$A_{5}$	$A_{6}$	$A_{7}$
BELLS2	2.78	0.912	−1.25	−0.428	0.553	2.63	0.027
BELLS3	0.95	0.892	−1.25	−0.448	0.621	1.83	0.042

**Table 2:** Mean Absolute Percentage Error for BELLS3 prediction.

Scenario\Case	5 cm	10 cm	15 cm	All
Winter	35.33	33.46	36.54	35.11
Summer	6.55	6.54	7.13	6.74

**Table 3:** Mean Absolute Percentage Error for NN prediction.

Scenario\Case	5 cm	10 cm	15 cm	All
Winter Test	2.65	2.71	2.99	2.78
Winter Validation	5.14	6.21	4.71	5.35
Summer Test	0.24	0.46	0.68	0.46
Summer Validation	0.35	0.41	0.53	0.43
